# Glatiramer Acetate (Copaxone) Modulates Platelet Activation and Inhibits Thrombin-Induced Calcium Influx: Possible Role of Copaxone in Targeting Platelets during Autoimmune Neuroinflammation

**DOI:** 10.1371/journal.pone.0096256

**Published:** 2014-05-02

**Authors:** Sarah C. Starossom, Tatyana Veremeyko, Marina Dukhinova, Amanda W. Y. Yung, Eugene D. Ponomarev

**Affiliations:** 1 Center for Neurologic Diseases, Brigham and Women's Hospital, Harvard Medical School, Boston, Massachusetts, United States of America; 2 Institute for Medical Immunology and NeuroCure, Charité - Universitätsmedizin Berlin, Berlin, Germany; 3 School of Biomedical Sciences, The Chinese University of Hong Kong, Shatin, NT, Hong Kong; University of Muenster, Germany

## Abstract

**Background:**

Glatiramer acetate (GA, Copaxone, Copolymer-1) is an FDA approved drug for the treatment of MS and it is very effective in suppressing neuroinflammation in experimental autoimmune encephalitis (EAE), an animal model of MS. Although this drug was designed to inhibit pathogenic T cells, the exact mechanism of EAE/MS suppression by GA is still not well understood. Previously we presented evidence that platelets become activated and promote neuroinflammation in EAE, suggesting a possible pathogenic role of platelets in MS and EAE. We hypothesized that GA could inhibit neuroinflammation by affecting not only immune cells but also platelets.

**Methodology/Principal Findings:**

We investigated the effect of GA on the activation of human platelets *in vitro*: calcium influx, platelet aggregation and expression of activation markers. Our results in human platelets were confirmed by *in-vitro* and *in-vivo* studies of modulation of functions of platelets in mouse model. We found that GA inhibited thrombin-induced calcium influx in human and mouse platelets. GA also decreased thrombin-induced CD31, CD62P, CD63, and active form of αIIbβ3 integrin surface expression and formation of platelet aggregates for both mouse and human platelets, and prolonged the bleeding time in mice by 2.7-fold. In addition, we found that GA decreased the extent of macrophage activation induced by co-culture of macrophages with platelets.

**Conclusions:**

GA inhibited the activation of platelets, which suggests a new mechanism of GA action in suppression of EAE/MS by targeting platelets and possibly preventing their interaction with immune cells such as macrophages. Furthermore, the reduction in platelet activation by GA may have additional cardiovascular benefits to prevent thrombosis.

## Introduction

Platelets play an important role in cardiovascular pathologies, but their role in neuroinflammatory diseases is not clear [1]–[3]. Recently it was demonstrated that platelets contributed to inflammation during rheumatoid arthritis and arthrosclerosis [4]–[6]. Activated platelets produce a number of pro-inflammatory mediators (cytokines, chemokines, histamin etc.) and could initiate and propagate inflammation [7]. It was demonstrated that platelets become activated during multiple sclerosis (MS) [8]. It was also reported that in MS patients there were increased numbers of platelet aggregates, platelet-derived microparticles and increased levels of the activation marker CD62P on the surface of platelets [9]. We and other group have demonstrated that the depletion of platelets substantially ameliorated central nervous system (CNS) autoimmune inflammation during experimental autoimmune encephalitis (EAE), an animal model of MS [10], [11]. Our previous study demonstrated that during EAE platelets become activated by sialated glycolipids integrated into neuronal and astroglial lipid rafts found in blood brain barrier structures, which was critical for the development of CNS autoimmune inflammation [11].

Currently IFN-β and glatiramer acetate (GA) are the most commonly used FDA-approved drugs for MS therapy [12]. Although it was well established that the cytokine IFN-β plays an immunomodulatory and regulatory role, much less is known about the mechanisms of actions of GA. Among the proposed mechanisms of GA action on the reduction of severity and frequency of MS relapses is the deactivation of myelin specific T cells and skewing CD4 T cells differentiation form pathological Th1 towards regulatory Th2 phenotypes [12]. In addition, it was proposed that GA affects innate immune cells including macrophages and dendritic cells [13], [14]. Particularly it was shown that GA influence monocyte/macrophage polarization by shifting the balance from pathological M1 towards the more regulatory M2 phenotypes [13]. Finally it was recently proposed that GA affected B cells [15], [16]. Despite the emerging evidence for great importance of platelets in MS pathophysiology, the possible action of GA on platelet functions has not been investigated so far.

It was shown that in several cases of treatment of MS patients, subcutaneous injections of IFN-β resulted in thrombosis in cutaneous venules leading to the formation of skin lesions [17]. Formation of skin lesions were also reported for GA injections [18], suggesting possible involvements of both drugs in modulation of platelet functions. Given the fact that MS patients often demonstrated other platelet abnormalities such as thrombocytopenia [19], we decided to investigate possible actions of GA and IFN-β on their capacity to modulate platelet functions.

In this study we found that GA, but not IFN-β, substantially inhibited thrombin-induced activation of human and mouse platelets, as was demonstrated by substantial reduction in the level of Ca^2+^ influx, a hallmark of platelet activation. Furthermore, our investigation demonstrated a strong inhibitory effect of GA on platelet activation as determined by the reduction in aggregate formation and a decrease in surface expression of CD62P and other platelet activation markers, which indicates a new mechanism of GA action during MS therapy.

## Results

### GA inhibites Ca^2+^ influx in human thrombin-activated platelets

**Figure 1:**
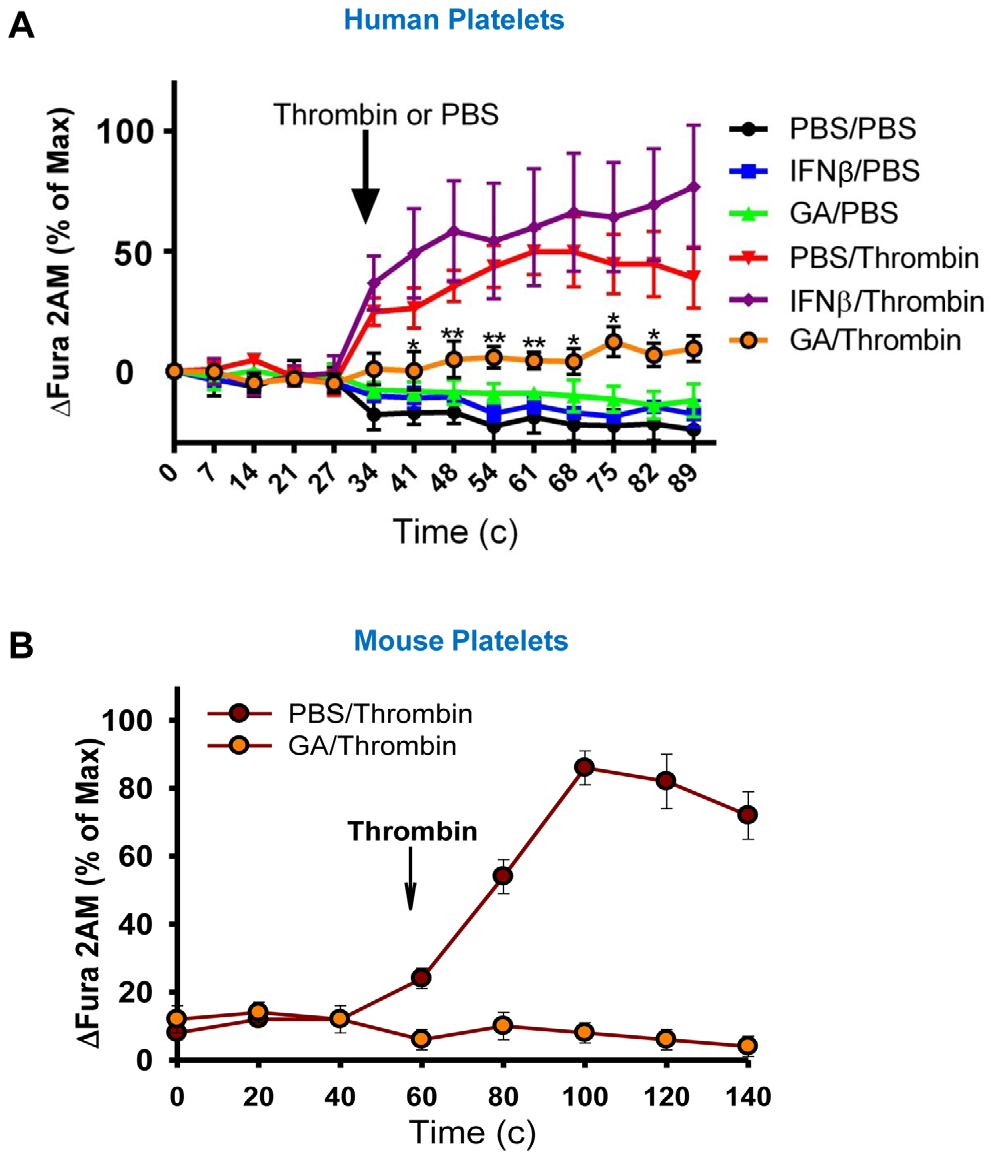
Analysis of effects of glatiramer acetate and IFN-β on thrombin-induced calcium influx in human and mouse platelets. Human (**A**) or mouse (**B**) platelets were isolated as described in *Methods* and pre-treated with glatiramer acetate (GA; 50–100 µg/ml), or IFN-β (100 U/ml), or PBS for 30 min, after which thrombin (0.1 U/ml) or PBS were added as indicated by arrow. Fluorescent probe Fura-2M was used to measure intracellular Ca^2+^ as described in *Methods*. (**A**) The relative level of changes in Fura-2M fluorescence (y-axis) vs. time (x-axis) is shown for human platelets pretreated with GA, IFN-β or PBS and then treated with thrombmin (*GA/Thrombin*, *IFNβ/Thrombin*, and *PBS/Thrombin*) or PBS (*GA/PBS*, *IFNβ/PBS*, and *PBS/PBS*). Mean ± S.E. of four separate experiments is shown. (*, p<0.05 and **, p<0.01 for *PBS/Thrombin* vs. *GA/Thrombin*). (**B**) The relative level of changes in Fura-2M fluorescence (y-axis) vs. time (x-axis) is shown for mouse platelets pretreated with GA or PBS and then activated with thrombmin. Mean ± S.E. of triplicate is shown.

In the first series of experiments we tested whether commonly used FDA-approved drugs for multiple sclerosis GA (Copaxone™) and IFN-β (Betaseron™) affected platelet functions as determined by measurement of thrombin-induced Ca2+ influx, an established hallmark of platelet activation. We investigated various doses of GA and IFN-β to determine whether they affect Ca2+ influx in thrombin-stimulated platelets isolated from normal healthy subjects. Calcium influx was measured by the calcium-specific fluorescence probe Fura-2M that detects intracellular concentration of Ca2+. We found that at optimal concentrations for GA (100 µg/ml) and IFN-β (100 U/ml), pretreatment of human platelets for 30 min with GA significantly decreased Ca2+ influx in thrombin-activated platelets (**Fig. 1A**; PBS/Thrombin and GA/Thrombin), whereas IFN-β enhanced it, although the increase was not statistically significant (**Fig. 1A**; PBS/Thrombin and IFNβ/Thrombin). In contrast to IFN-β, the effect of the inhibition of Ca2+ influx in thrombin-activated platelets by GA was statistically significant for all measured time-points (**Fig. 1A**; PBS/Thrombin and GA/Thrombin). Inhibition of Ca2+ influx was observed for the range of concentrations of GA from 20 to 200 µg/ml with the strongest effect for concentration of 50–100 µg/ml (not shown). Thus, GA strongly affected functions of human platelets by inhibiting thrombin-induced Ca2+ influx. We also tested whether GA affects functions of mouse platelets and found that GA inhibited Ca2+ influx in thrombin-activated mouse platelets as well (**Fig. 1B**). This indicates that GA inhibits thrombin-induced Ca2+ influx in both human and mouse platelets.

### GA inhibits upregulation of CD62P and other activation markers in human and mouse thrombin-activated platelets

**Figure 2:**
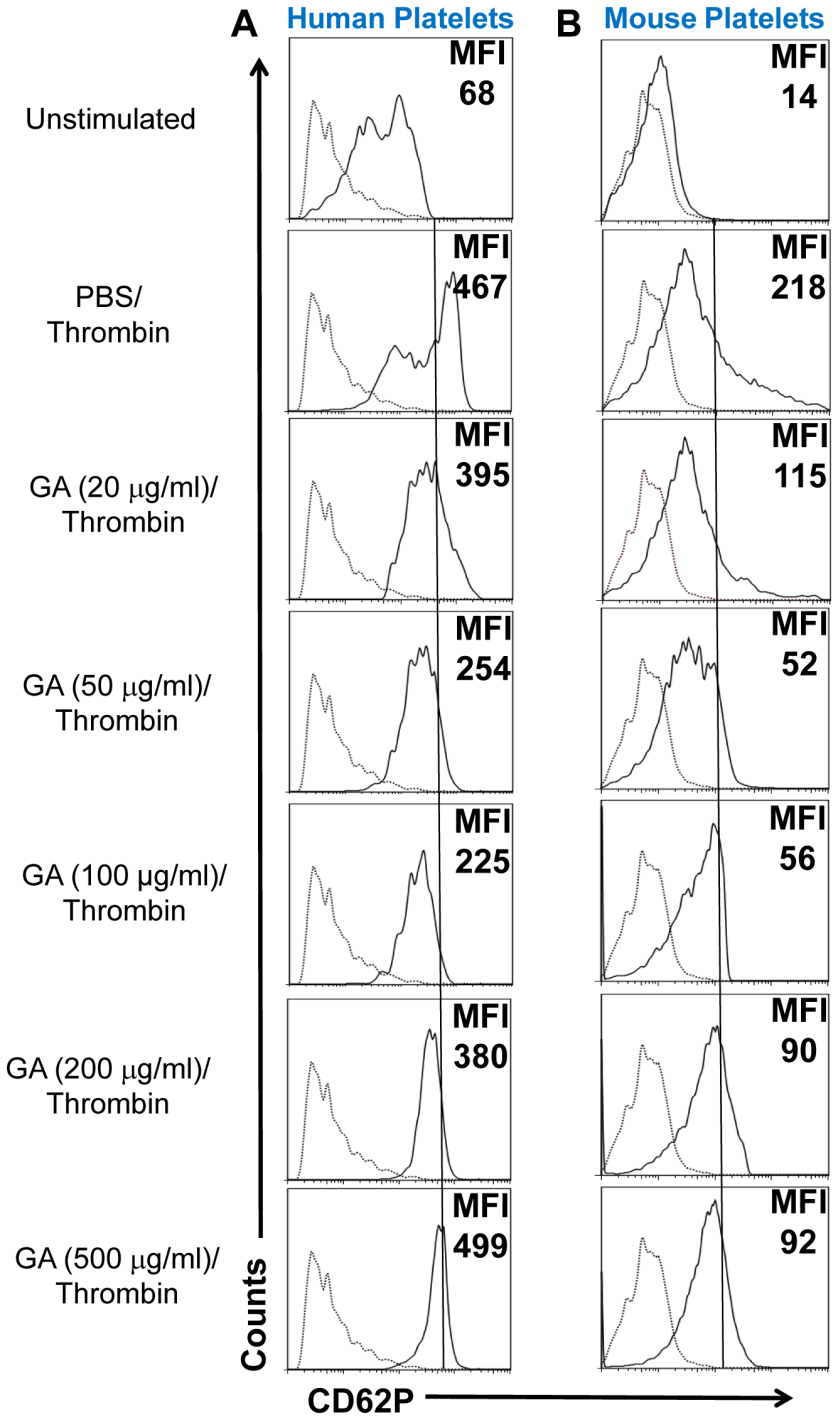
Effect of glatiramer acetate (GA) on thrombin-induced surface expression of CD62P in human and mouse platelets. Human (**A**) or mouse (**B**) platelets were isolated as described in *Methods* and were pretreated with PBS or various concentrations of glatiramer acetate (GA; 20–500 µg/ml) for 30 min. After pretreatment with GA, platelets were activated with thrombin and then analyzed by FACS as described in *Methods*. Histograms for CD62P (solid lines) or isotype control (dotted lines) expressions are shown for CD42a^+^CD61^+^ gated human (**A**) or CD41^+^CD61^+^ gated mouse (**B**) platelets. Mean fluorescent intensity (MFI) for CD62P expression is shown in upper right corner of each histogram. Representative experiment of five is shown.

**Figure 3:**
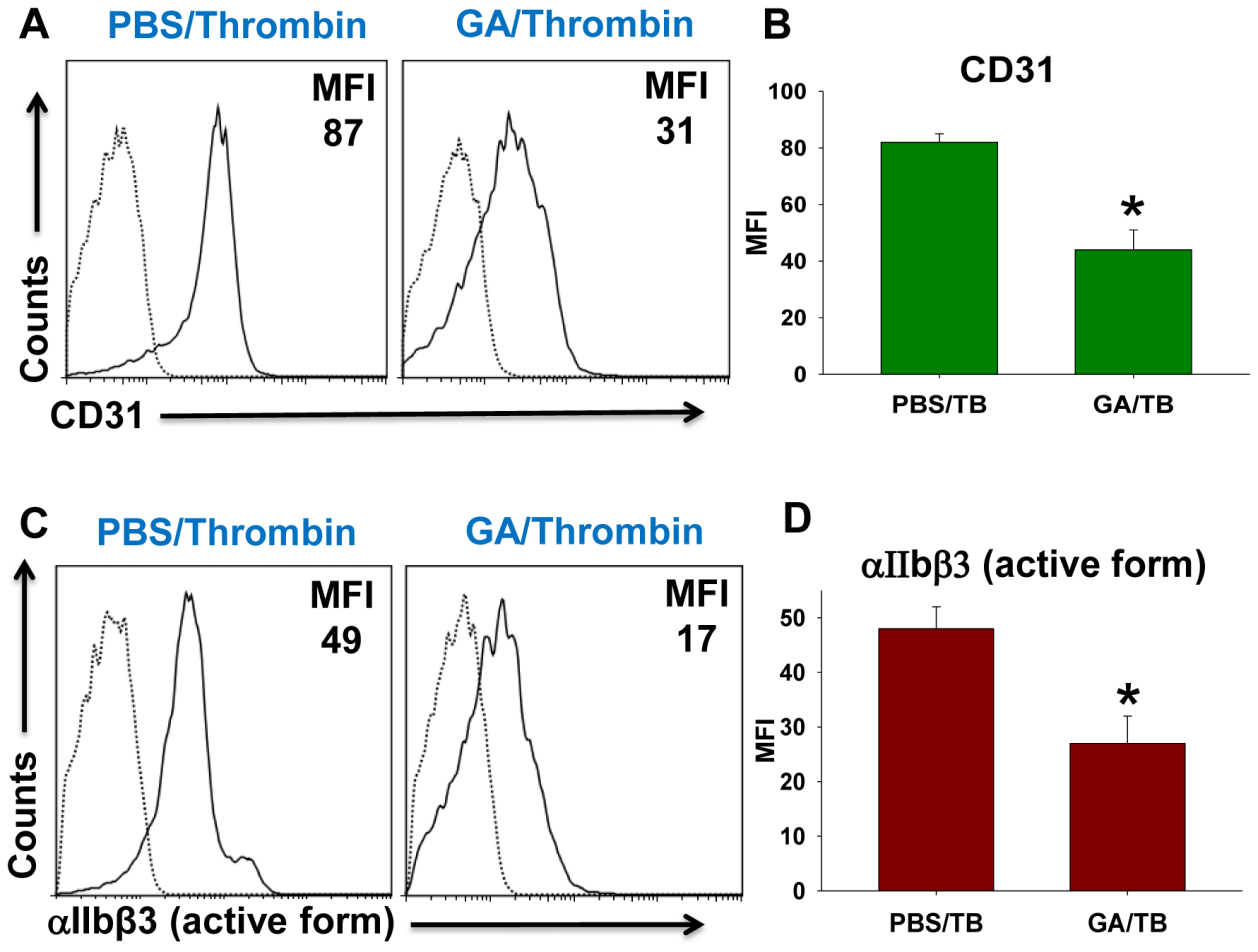
Effect of glatiramer acetate (GA) on thrombin-induced expression of activation marker CD31 and active form of αIIbβ3 integrin on the surface of mouse platelets. Mouse platelets were isolated and pretreated with PBS or GA (50 µg/ml) for 30 min. After pretreatment with PBS or GA, platelets were activated with thrombin and then analyzed for the expression of activation markers by three-color flow cytometry as for Fig.2. (A, C) Histograms for CD31 (A, solid lines), or active form of αIIbβ3 integrin (B, solid lines), or proper isotype control (dotted lines) expressions are shown for CD41^+^CD61^+^ gated mouse platelets. Mean fluorescent intensity (MFI) of activation marker expression is shown in upper right corner of each histogram. Mean ± S.E. of four separate experiments is shown in (B) and (D) (*, p<0.05).

We further investigated how GA affects thrombin-induced platelet activation in human and mouse platelets *in vitro*. In addition to calcium signaling, it was shown that activated platelets upregulated CD62P on their surface, which was investigated by flow cytometry. We found that the baseline level of CD62P in human platelets varied (with up-to 8-fold difference for mean fluorescence intensity (MFI) levels) among different examined subjects (N = 17; not shown) and was typically from low to intermediate levels as shown in one of the representative experiments (**Fig 2B**; *Unstimulated*). Platelets form unmanipulated mice had low level of CD62P (**Fig. 2A**; *Unstimulated*), demonstrating their non-activated status. Activation of platelets with thrombin induced upregulation of CD62P in both human and mouse platelets (**Fig. 2A** and **Fig. 2B**; *PBS/Thrombin*). However pre-treatment of human or mouse platelets with GA at concentration 20–200 µg/ml diminished CD62P upregulation with the strongest effect of 100 µg/ml for human and 50 µg/ml for mouse platelets (**Fig. 2A** and **Fig. 2B**; *GA(20*–*200* µ*g/ml*)*/Thrombin*), which was consistent with our data on Ca^2+^ influx. The inhibitions of CD62P expression at optimal doses (50 and 100 µg/ml) were statistically significant for both, human and mouse platelets (**Table 1**, *CD62P*). At the concentration of 500 µg/ml or higher, GA had little/no effect or even enhanced CD62P expression, suggesting that at a high doses of GA could activate platelets by increasing CD62P expression (**Fig. 2A** and **Fig. 2B**; *GA(500* µ*g/ml*)*/Thrombin*). Thus we found that at the optimal range of concentrations, GA inhibited platelet activation as determined by CD62P expression level; however at a very high concentrations (>500 µg/ml) GA increased platelet activation as determined by upregulation of CD62P. We further investigated expression of other activation markers for human and mouse platelets. We found that at concentration of 50–100 µg/ml GA decreased MFI levels of active form of αIIbβ3 integrin and CD63 by 43±5% and 57±4%, respectively for thrombin-activated human platelets (**Fig. S1**), and decreased ∼2-fold MFI levels of active form αIIbβ3 integrin and CD31 for thrombin-activated mouse platelets (**Fig. 3**). Thus, GA decreased expression of all examined activation markers on the surface of thrombin-activated human and mouse platelets.

**Table 1:** Statistical analysis of effects of glatiramer acetate (GA) on thrombin-induced CD62P upregulation and platelet aggregate formation for human and mouse platelets^1^.

	Human Platelets		Mouse Platelets	
	Control (PBS)	Glatiramer acetate^2^	Control (PBS)	Glatiramer acetate^3^
CD62P (MFI)^4^	378±24	244±9^5^***	123±32	42±5^6^*
Aggregates (%)^7^	18±2	8±1^8^**	21±3	7±2^8^**

1 Human or mouse platelets were stimulated with thrombin in the presence of GA vs. Control (PBS) as described in *Methods*.

2 Optimal concentration of GA is 100 µg/ml for human platelets.

3 Optimal concentration of GA is 50 µg/ml for mouse platelets.

4 Mean fluorescence intensity (MFI) of CD62P expression on the surface of CD42a^+^CD61^+^ gated human or CD41^+^CD61^+^ gated mouse platelets was analyzed by FACS as described *in Methods*. Mean ± S.E. of five separate experiments is shown.

5 ***, P<0.001.

6 *, P<0.05.

7 Percentages (%) of FCS^hi^SSC^hi^ aggregated CD42a^+^CD61^+^ gated human or CD41^+^CD61^+^ mouse platelets was analyzed by FACS as described in *Methods*. Mean ± S.E. of five separate experiments is shown.

8 **, P<0.01.

### GA inhibits aggregate formation of human and mouse thrombin-activated platelets

**Figure 4:**
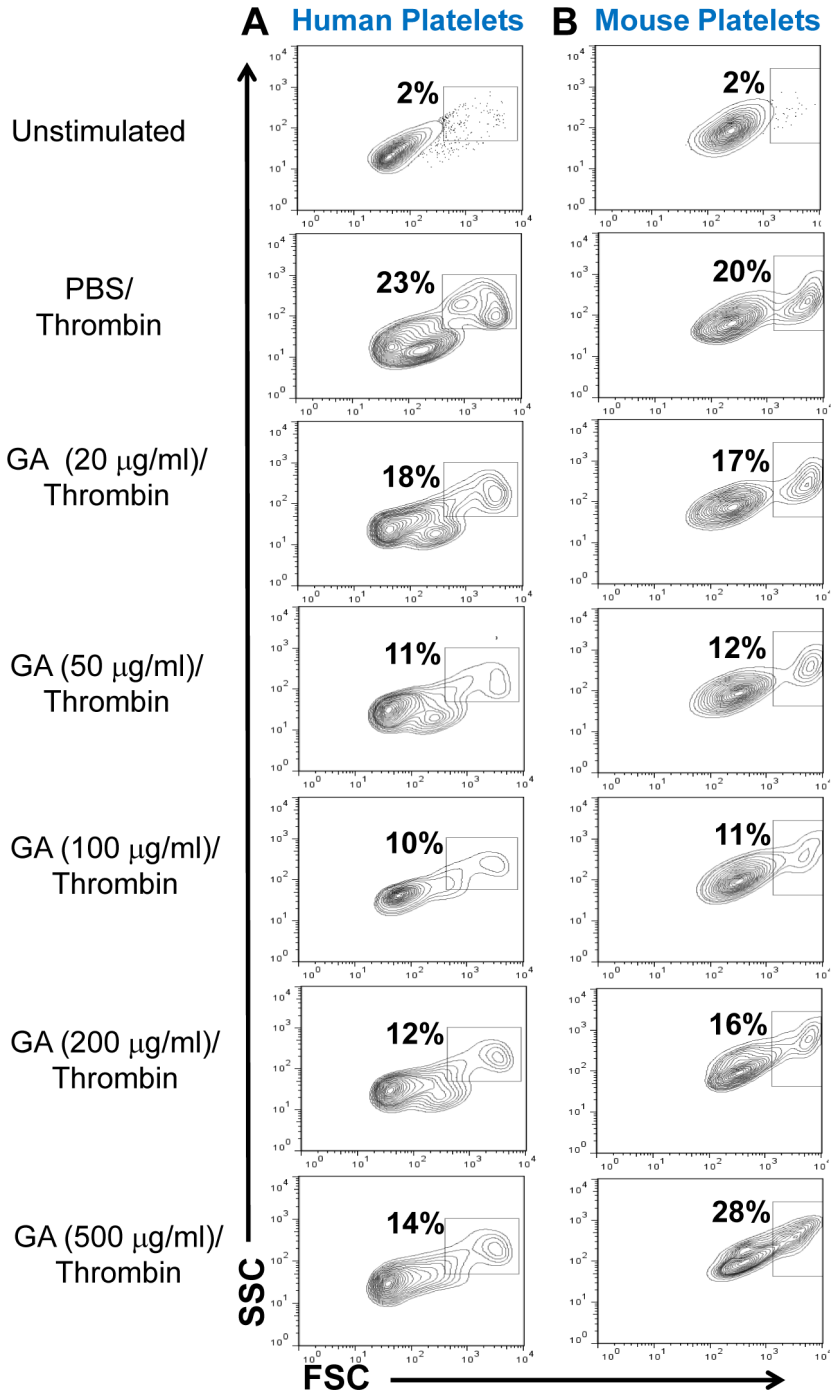
Effect of glatiramer acetate (GA) on thrombin-induced platelet aggregate formation. Human (**A**) or mouse (**B**) platelets were isolated as described in *Methods* and were pretreated with PBS or various concentrations of GA (20–500 µg/ml) for 30 min. After pretreatment with GA, platelets were activated with thrombin and then analyzed by FACS for aggregate formation as described in *Methods*. Contour plots for forward scatter (FSC; x-axes) vs. side scatter (SSC; y-axes) parameters are shown for CD42a^+^CD61^+^ gated human (**A**) or CD41^+^CD61^+^ gated mouse (**B**) platelets. Percentages of FCS^hi^SSC^hi^ aggregated platelets are shown on a top of square gates of contour plots. Representative experiment of five is shown.

**Figure 5:**
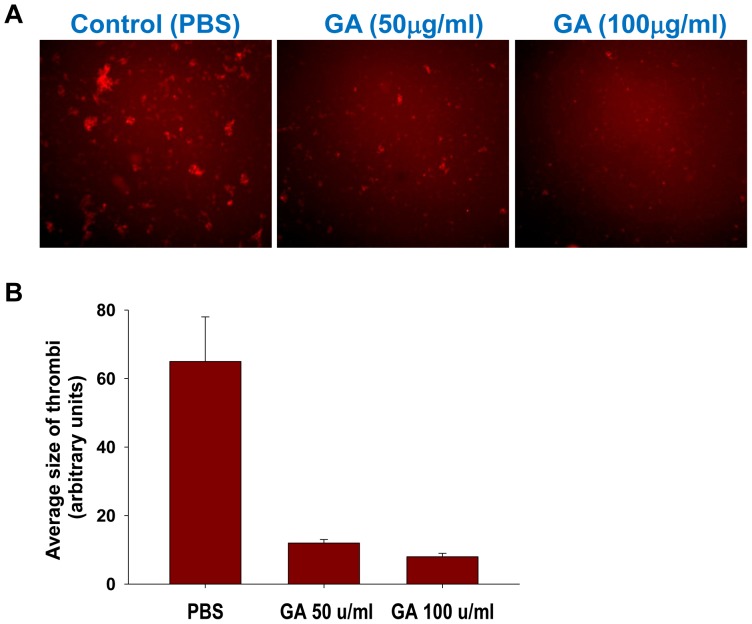
Effect of glatiramer acetate (GA) on platelet adhesion to collagen and aggregation under flow conditions. Whole mouse blood samples were collected from normal C57BL/6 mice using sodium citrate as anticoagulant and pretreated with PBS or GA (50 or 100 µg/ml) for 30 min as described in *Methods*. After pretreatment with PBS or GA, the whole blood samples were labeled with platelet-specific antibodies and then perfused through the camber with collagen coated glace slides as described in *Methods*. Collagen coated slides with aggregated pre-labeled platelets were washed, fixed and subsequently analyzed using fluorescence microscopy. Representative immunofluorescent images for samples treated with PBS or GA (50 or 100 µg/ml) are shown in (**A**). Average size of thrombi ± S.E. is shown in (**B**).

Next we investigated the effect of GA on thrombin-induced platelet aggregate formation. Similarly to CD62P expression, GA decreased the percentages of aggregated platelets with the strongest effect at concentrations of 100 µg/ml for human (**Fig. 4A**) and 50 µg/ml for mouse palettes (**Fig. 4B**). At the high concentrations (500 µg/ml), the percentage of aggregated platelets was even increased in case of mouse platelets (**Fig. 4B**). The increase in aggregate formation by very high concentrations of GA was again consistent with CD62P expression (**Fig. 2**). The inhibition of platelet aggregate formation at optimal concentrations of 50 and100 µg/ml were statistically significant for mouse and human platelets, respectively (**Table 1**, *Aggregates*). Thus at concentrations of 50–200 µg/ml GA decreased platelet activation as determined by aggregate formation. Similarly to CD62P expression, high concentrations of GA (>500 µg/ml) increased platelet aggregation. We confirmed inhibitory effect of GA on platelet aggregate formation in another assay where platelets adhered to collagen and aggregated under flow conditions. At concentration of 50 and 100 µg/ml, GA substantially decreased formation of platelet aggregates under flow conditions by decreasing 6–8-fold the average size of thrombi (**Fig. 5**).

Finally we investigated whether IFN-β affects expression of CD62P on the surface of thrombin-activated platelets and their aggregate formation. We found that at the concentration of 100U/ml IFN-β enhanced thrombin-induced expression of CD62P (**Fig. S2**), but did not affect platelet aggregate formation as determined by FACS (not shown). Thus we found that GA, but not IFN-β, inhibited platelet functions as determined by decrease in expression of activation markers and reduction in aggregate formation.

### GA affects interaction of platelets with macrophages *in vitro*

**Figure 6:**
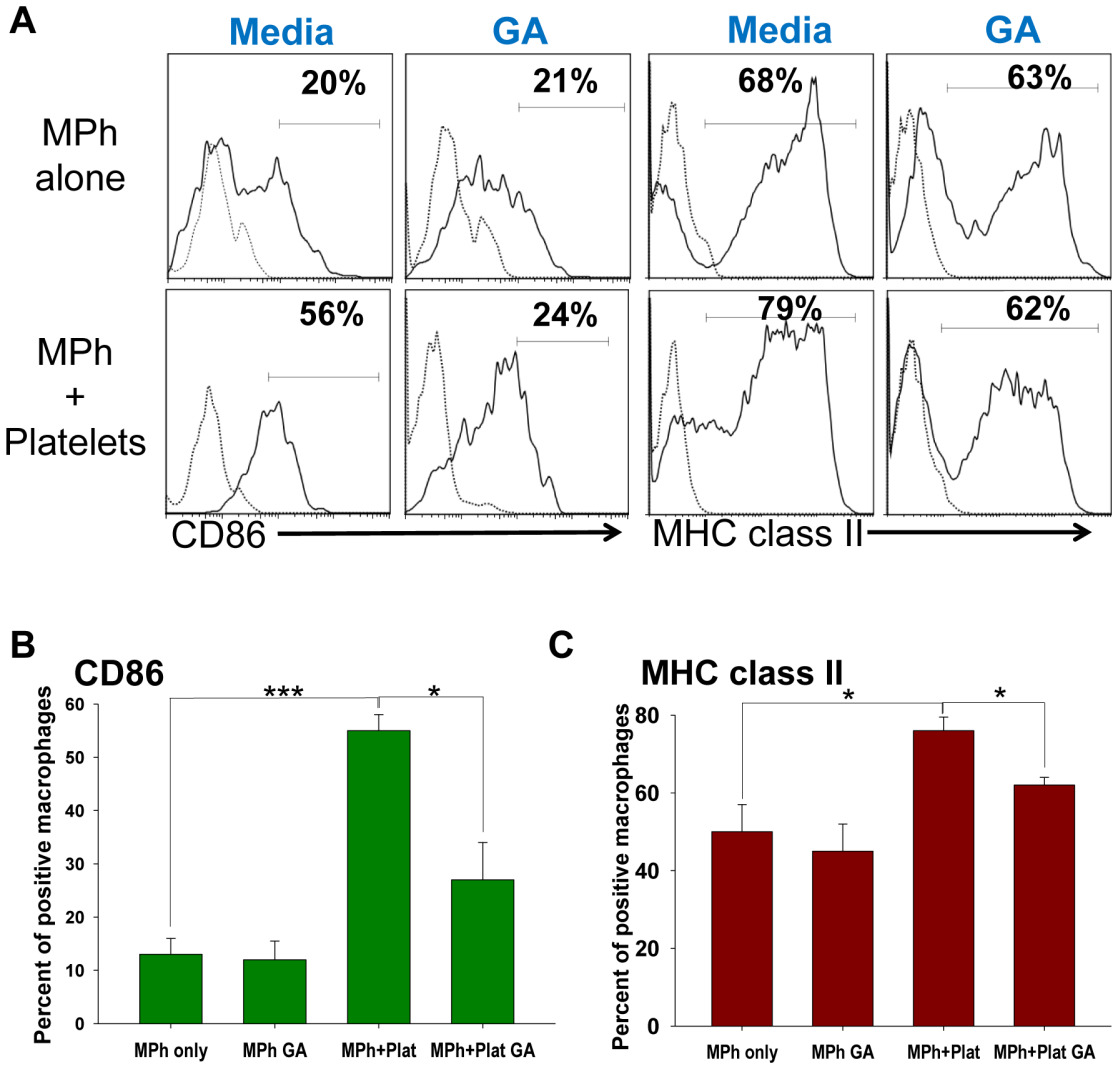
The role of glatiramer acetate (GA) in platelet-dependent activation of macrophages *in vitro*. Mouse peritoneal macrophages were isolated and cultured alone or co-cultured with autologous platelets with or without glatiramer acetate. After 24 hours of single culture or co-culture with platelets, macrophages were stained for CD11b, F4/80, CD86 and MHC class II and analyzed by four-color flow cytometry as described in *Methods.* (**A**) Histograms for CD86 or MHC class II (solid lines) or proper isotype control (dotted lines) expressions are shown for CD11b^+^F/80^+^ gated macrophages. Percentages of positive cells are shown on the top of each histogram (**B, C**) Mean ± S.E. of three experiments is shown (*, p<0.05 and ***, p<0.001). *Abbreviations*: MPh, macrophages; GA, glatiramer acetate, Plat, platelets.

We and others have found that depletion of platelets ameliorated neuroinflammation in animal model of MS [10], [11]. It was also proposed that platelets could interact with macrophages and promote their activation and migration to the site of inflammation [20], [21]. Based on this, it was hypothesized that interaction of platelets with immune cells such as macrophages is one of the major mechanisms of initiation and perpetuation of neuroinflammation by platelets [10], [11]. To test whether GA affect interaction of platelets with immune cells such as macrophages, we co-cultured macrophages with autologous platelets in the presence of GA. We found that platelets contributed macrophage activation *in vitro* as determined by upregulation of two examined macrophage activation markers: co-stimulatory molecule CD86 and MHC class II (**Fig. 6**). Addition of GA to co-cultured macrophages and platelets significantly decreased platelet-induced upregulation of CD86 and MHC class II on macrophages. Thus, GA decreased platelet-mediated activation of macrophages.

### GA prolongs bleeding time *in vivo*

**Figure 7:**
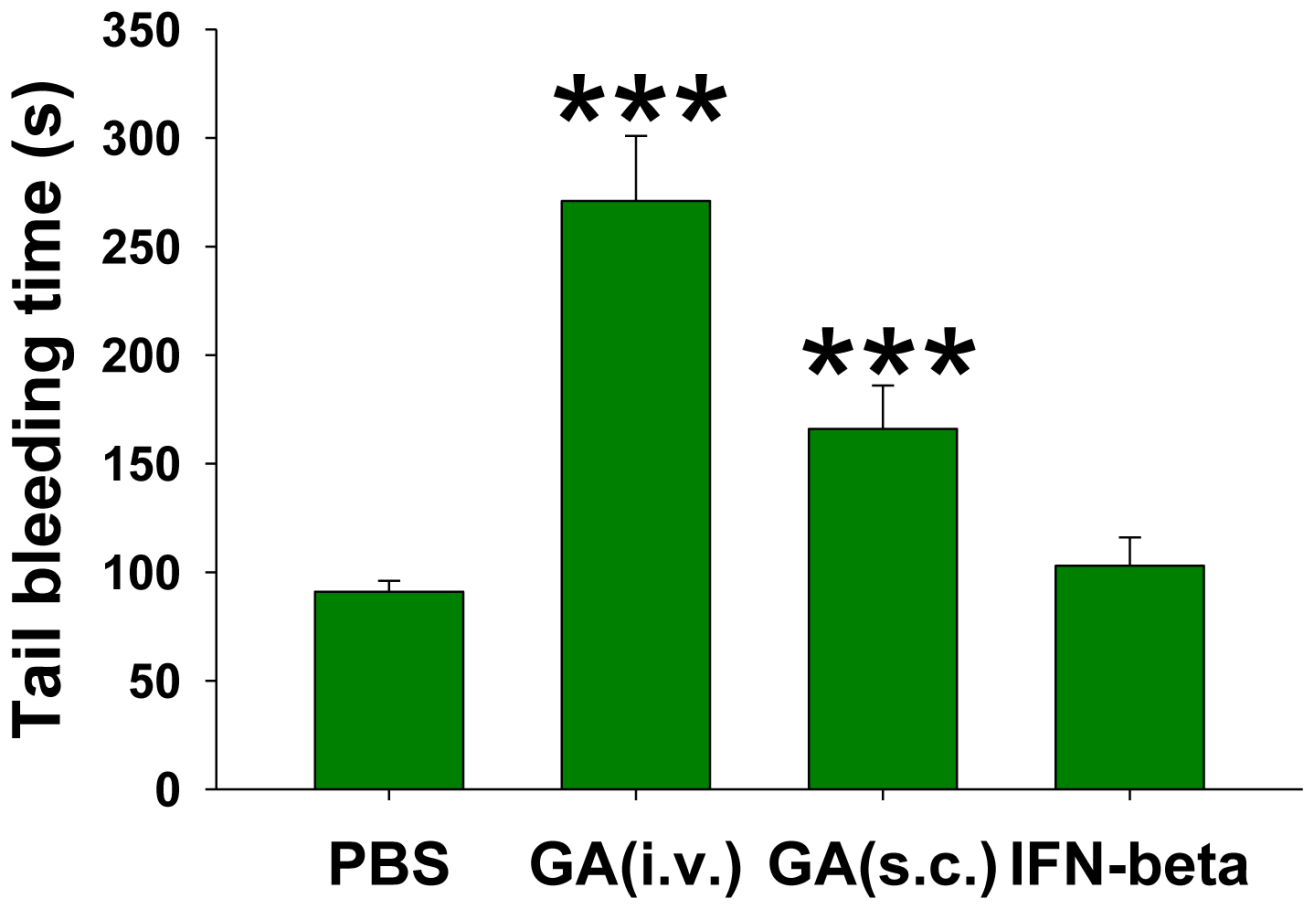
Analysis of bleeding time in mice after administration of glatiramer acetate. Mice were administrated intravenously or subcutaneously with GA, or IFN-β, or PBS, and 30–60 minutes later, tail bleeding test was performed as described in *Methods*. Duration of bleeding (y-axis, seconds) is shown. Mean ± S.E. of total 9–12 individual animal in each group of three separate experiments is shown (***, p<0.001).

Since we found that GA had a similar inhibitory effect on both human (**Fig. 2A** and **Fig. 4A**) and mouse (**Fig. 2B** and **Fig. 4B**) platelets, we further tested the effect of GA on murine platelets *in vivo*. One of the commonly used rodent models to test the platelet functions *in vivo* is the tail bleeding test [22]. If the activation of platelets is inhibited, bleeding time increases as we described in our previous study [11]. Indeed we found that i.v. and s.c. administration of GA (see *Methods*) significantly increased the bleeding time, suggesting an inhibitory effect of GA on platelet activation *in vivo*. At the same time i.v. administration of IFN-β did not have any statistically significant effects on tail bleeding time (**Fig. 7**). Thus GA, but not IFN-β, inhibited platelet activation *in vivo* in mouse model of tail bleeding.

## Discussion

In this study we investigated the effect of GA on platelet physiology *in vitro* and *in vivo*. We found that GA modulates platelet functions by decreasing their level of activation and thus leading to an increased bleeding time *in vivo*. This observation is of particular importance, as the exact mechanisms of therapeutic effect of GA in MS are still not fully understood and the effects of GA on platelets have not been reported yet.

Previously we found that platelets substantially contributed to neuroinflammation in the EAE model [11]. Giving the fact that GA effectively suppressed EAE [12], one potential mechanism of GA action on reduction of EAE severity and possibly MS is by decreasing platelet activation and their communication with leukocytes.

We believe that the effect of GA on platelets is beneficial for MS, since platelets play a pathogenic role in EAE model [10], [11]. Also GA might have a beneficial cardiovascular effect by reducing thrombosis. Similarly to our findings with GA, Nakano et al. showed that estrogen decreased the Ca^2+^ influx in thrombin-induced platelets [23], and suggested a beneficial role for hormonal replacement therapy on the prophylaxis of thrombosis in post-menopause women [23].

Apart from possible beneficial effects, GA action on platelets especially at high doses (more than 0.5 mg/ml) could explain some of the side effects of GA injections. For example, necrotic lesions with the signs of vein thrombosis were found at the sites of injections in both IFN-β and for GA treatment [17], [18]. This goes along with our observation that IFN-β (at concentration of 100 U/ml) and GA (at high concentrations, >0.5 mg/ml) increased platelet activation. It was also demonstrated that GA may cause specific symptoms such as chest pain, flushing, dyspnea, and anxiety [24]. As we demonstrated previously in mouse model, dyspnea and restless behavior are both associated with massive platelet activation/degranulation and associated with the release of potent mediators from platelets such as histamine [11]. Other investigators found that in humans massive platelet activation/degranulatuion is caused by the administration of radiographic contrast media into blood vessels, indicating that human platelets may degranulate in response to the injection of chemical substances [25]. According to our study, we believe that platelets should not be exposed to high concentrations of GA in order to decrease side effects of GA injections.

Taken together, our study demonstrated a new possible mechanism of suppression of CNS autoimmune inflammation by GA via targeting platelet activation. We believe that the results of our study will allow a better understanding of the therapeutic effects of GA, with an important implication of improving the effectiveness of this drug and reducing its side effects during treatment of MS.

## Conclusions

The findings in this study demonstrated that GA suppresses platelet activation. These results suggest a new possible mechanism of EAE/MS suppression by GA by targeting platelets. Although it remains unclear whether the effect of GA on platelets plays a primary or secondary roles role in MS therapy when compared to other GA-affected cell types such as lymphocytes, this study provides a new application for GA to modulate platelet functions during neuroinflammation or other pathologies.

## Materials and Methods

### Antibodies and reagents

Thrombin was purchased for Sigma. Human IFN-β was purchased from PBL Interferon Source. Mouse IFN-β was purchased from R&D Inc. Anti-human CD42a-PeCP, CD62P-FITC, CD63-PE, and FITC conjugated PAC-1 antibodies (recognize active form of human αIIbβ3 integrin) were purchased form BD Biosciences. Anti-mouse CD31-PE, CD41-PE, CD61-FITC, CD62P-Biotin, CD86-PE antibodies, and Streptavidin-APC were purchased form BD Biosciences. Anti-mouse F4/80-APC and CD41-APC antibodies were purchased from eBiosciences. Anti-mouse CD11b-AF488, and MHC class II-PE-Cy5 antibodies were purchased from Biolegend. PE conjugated JON/A antibodies (recognize active form of mouse αIIbβ3 integrin) and fluorescently labeled anti-CD42c antibodies for in vivo labeling of mouse platelets were purchased from Emfret Analytics Inc. Fura-2M probe, a membrane-permeable derivative of Fura-2, was purchased from Molecular Probes. GA (Copaxone™) was purchased from Teva. Undiluted Copaxone (20 mg/ml) was stored at +4°C. For experiments GA was diluted to proper concentrations in PBS, stored at room temperature and used within 12 hours.

### Platelet isolation and analysis of activation

For the isolation and analysis of platelets, peripheral blood samples were drawn using 10 ml collecting tubes with EDTA (BD Biosciences) from healthy control subjects as described in our previous study [11]. Mouse platelets were isolated from C57BL/6 (B6) mice as described previously [11]. The analysis of platelet surface marker expression and Ca^2+^ influx was preceded by the pretreatment with GA or IFN-β for 30 min and activation with thrombin and was performed and presented as described in details in our previous work [11] and the work by Nakano et al [23]. Blood samples were kept at room temperature and platelets were analyzed within 1–3 hours after blood collection.

### Platelet aggregation

For analysis of platelet aggregation by FACS, mouse or human platelets were incubated with PBS or Copaxone for 30 min, activated with thrombin (0.1 U/ml), and stained with anti-CD61 and anti-CD41 antibodies (mouse), or anti-CD42a and anti-CD61 antibodies (human) as described [11]. The samples were analyzed by two-color flow cytometry. Gated CD42a^+^CD61^+^ human or CD41^+^CD61^+^ mouse platelets were analyzed for the forward/side scatter parameters as indicators of the platelet shape change and aggregation as described earlier [11]. Analysis of platelet aggregation under flow conditions was performed as originally described [26]–[28] with minor modifications. Mouse blood was collected using sodium citrate as anticoagulant, whole blood samples were incubated with PBS or with Copaxone for 30 min at room temperature, then platelets were pre-labeled with antibodies for in vivo mouse platelet labeling (Emfret) at room temperature for 15 min. After which, the blood samples were perfused through the flow chamber with slides coated with mouse fibrillar collagen at a shear rate of 1200 s^−1^ for 5 minutes. After perfusion, the slides were removed from the camera, thoroughly washed with PBS, fixed with PBS with 1% paraformaldehyde overnight and analyzed by fluorescent microscopy. Relative sizes of thrombi were measured using ImageJ software.

### Co-culture of platelets with macrophages

Peritoneal macrophages were isolated from C57BL/6 mice as we described in our previous experiments [29] and were cultured alone (0.5×10^6^/well in 24-well plate) or co-cultured with autologous platelets at the ratio of 1∶20 for 24 hours with or without Copaxone (50 µg/ml). After 24 hours of single culture or co-culture with platelets, macrophages were washed and stained for CD11b, F4/80, CD86 and MHC class II similarly as described [29] and analyzed by 4-color cytometry. CD11b^+^F4/80^+^ gated macrophages were analyzed for the expression of CD86 and MHC class II. Fc receptors on macrophages were blocked using anti-FcR blocking antibodies to avoid non-specific labeling (BD Biosceneces).

### Analysis of mouse tail bleeding

C57BL/6 mice were purchased from Jackson Laboratories. For the assessment of platelet activation *in vivo*, mice were injected with GA i.v. (200 µl/mouse 3.5 mg/kg for i.v., and 400 µl/mouse 12.5 mg/kg for s.c. injections) and 30 min (for i.v.) or 60 min (for s.c.) later the tail was cut approximately 5 mm above the tail end and immersed into saline similarly as described in our previous study to determine the duration of the bleeding period [11]. IFN-β was administered i.v. at a dose of 2×10^4^ U per mouse. The optimal doses of administrated drugs were established in the preliminary experiments as effective and well tolerated by animals.

### Ethics

Use of human platelets from peripheral blood samples of anonymous healthy individuals for *in vitro* experiments was approved by the institutional review boards of the Brigham and Women's Hospital and all healthy subjects provided written informed consent prior to enrollment into study. The data from human blood samples was obtained and analyzed without records of any personal information to ensure complete anonymity. All animal protocols were approved by the Harvard Medical School Institutional Animal Care and Use Committee and Chinese University of Hong Kong Animal Ethics Committee. For experiments that involved pain and distress the appropriate anesthetic and analgesic drugs were used. Euthanasia was performed by using carbon dioxide.

### Statistical analysis

Student's t-test and Mann Whitney u-test were used to validate the significance of the observed differences. A p-value of less than 0.05 was considered statistically significant.

## Supporting Information

Figure S1 **Effect of glatiramer acetate (GA) on thrombin-induced expression of activation marker CD63 and active form of αIIbβ3 on the surface of human platelets.** Human platelets were isolated and pretreated with PBS or glatiramer acetate (100 µg/ml) for 30 min ad described in *Methods*. After pretreatment with GA, platelets were activated with thrombin (0.1 U/ml) and then analyzed by three-color flow cytometry as for Fig.2. Representative histograms of expressions of active form of αIIbβ3 integrin (left histograms; solid lines) or CD63 (right histograms; solid lines) or proper isotype controls (dotted lines) are shown for CD42a^+^CD61^+^ gated human platelets.
(TIF)

Figure S2 **Effect of IFN-β on thrombin-induced surface expression of CD62P in mouse platelets.** Mouse platelets were isolated as described in *Methods* and were pretreated with PBS or IFN-β (100 U/ml) for 30 min. After pretreatment with PBS or IFN-β, platelets were activated with thrombin and then analyzed by FACS as for Fig. 2. (**A**) Histograms for CD62P (solid lines) or isotype control (dotted lines) expressions are shown for CD41^+^CD61^+^ gated mouse platelets. Mean fluorescent intensity (MFI) for CD62P expression is shown in upper right corner of each histogram. (**B**) Mean ± S.E. of four separate experiments is shown (*, p<0.05).
(TIF)
