# Supplementary figures and images for: Glatiramer Acetate (Copaxone) Modulates Platelet Activation and Inhibits Thrombin-Induced Calcium Influx: Possible Role of Copaxone in Targeting Platelets during Autoimmune Neuroinflammation
Source: PLoS One. 2014 May 2;9(5):e96256. doi: 10.1371/journal.pone.0096256 (PMC4008572; doi:10.1371/journal.pone.0096256)

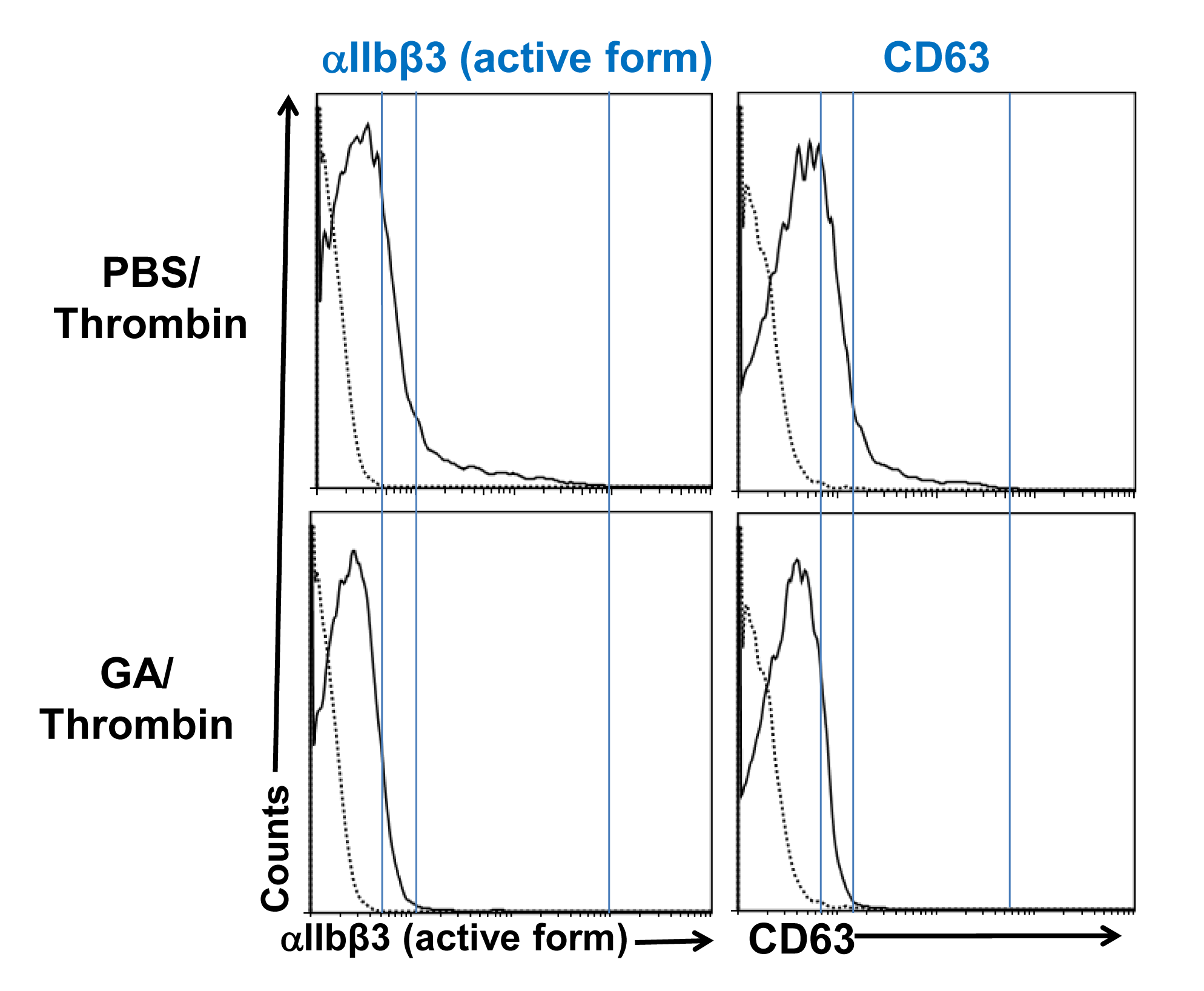

Supplement: Figure S1 — Effect of glatiramer acetate (GA) on thrombin-induced expression of activation marker CD63 and active form of αIIbβ3 on the surface of human platelets. Human platelets were isolated and pretreated with PBS or glatiramer acetate (100 µg/ml) for 30 min ad described in Methods . After pretreatment with GA, platelets were activated with thrombin (0.1 U/ml) and then analyzed by three-color flow cytometry as for Fig.2. Representative histograms of expressions of active form of αIIbβ3 integrin (left histograms; solid lines) or CD63 (right histograms; solid lines) or proper isotype controls (dotted lines) are shown for CD42a+CD61+ gated human platelets. (TIF) [file pone.0096256.s001.tif]

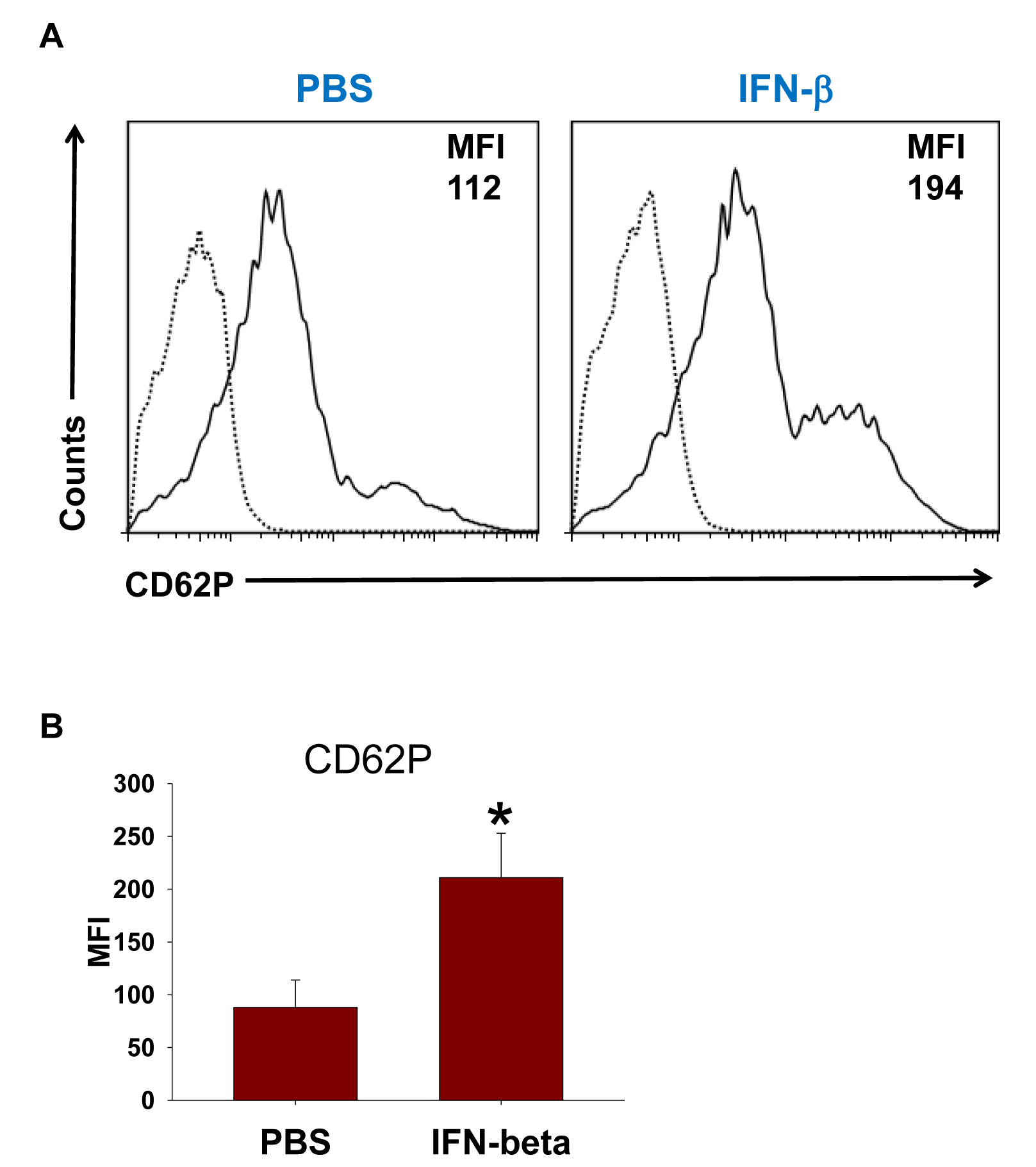

Supplement: Figure S2 — Effect of IFN-β on thrombin-induced surface expression of CD62P in mouse platelets. Mouse platelets were isolated as described in Methods and were pretreated with PBS or IFN-β (100 U/ml) for 30 min. After pretreatment with PBS or IFN-β, platelets were activated with thrombin and then analyzed by FACS as for Fig. 2. (A) Histograms for CD62P (solid lines) or isotype control (dotted lines) expressions are shown for CD41+CD61+ gated mouse platelets. Mean fluorescent intensity (MFI) for CD62P expression is shown in upper right corner of each histogram. (B) Mean ± S.E. of four separate experiments is shown (*, p<0.05). (TIF) [file pone.0096256.s002.tif]
